# Aging and Longevity between Genetic Background and Lifestyle Intervention

**DOI:** 10.1155/2014/516402

**Published:** 2014-05-27

**Authors:** Giuseppe Passarino, Giuseppina Rose, Dina Bellizzi, Maria De Luca, Efstathios S. Gonos

**Affiliations:** ^1^Department of Biology, Ecology and Earth Science, University of Calabria, 87036 Rende, Italy; ^2^Department of Nutrition Sciences, University of Alabama at Birmingham, Birmingham, AL 35294, USA; ^3^Institute of Biology, Medicinal Chemistry and Biotechnology, National Hellenic Research Foundation, 11635 Athens, Greece

The search for the genetic and molecular basis of aging and longevity has blossomed over the past few decades. Many (correctly, in our opinion) consider that this scientific field started with the experiments of Tom Johnson in the 80s of the last century. Indeed, before then, most gerontologists not only proclaimed the lack of progress in the field, but also suggested that progress in the field was not possible because aging is ineluctable. In their view, aging occurs after reproduction, and then there is no need and also no opportunity for selection to act on genes that are expressed late in life. The analysis of hybrids obtained from different strains of* C. elegans* allowed estimating the heritability of life-span to be between 20% and 50%. In addition, Johnson found that mutations in a specific gene, named* Age1*, were able to significantly increase lifespan. These experiments triggered a number of genetic studies in both humans and model organisms aimed at identifying the genes and the biochemical pathways that can modulate lifespan. This fruitful quest led to the identification of genes strictly correlated with the maintenance of the cell and of its basic metabolism. Indeed, mutations in genes encoding proteins involved in DNA repair, telomere conservation, heat shock response, and the management of free radicals' levels were found to contribute to longevity or, in case of reduced functionality, to accelerated senescence (cellular aging) and the consequent organism aging. Concurrent efforts also showed that genes implicated in lipoprotein metabolism (especially APOE), immunity, and inflammation play a role in aging, age-related disorders, and organism longevity. Overall, these observations led to the idea that longevity may arise from a particularly efficient process of maintenance of the cellular and organismal activities that could contrast the inevitable time related decline of the organism functionality, which in turn leads to death.

In parallel to the studies mentioned above, a substantial number of findings in model organisms suggested that longevity could “directly” be promoted by some specific pathways. These studies were mostly driven by the finding that calorie restriction (which is a reduction in nutrient intake in the absence of malnutrition) leads to a significant lifespan increase in a variety of organisms. The identification of pathways involved in the molecular mechanisms modulated by calorie restriction has shown that this dietary regime induces an arrest of the start of cell division; consequently, the cell enters in a quiescent status that prolongs its lifespan. In addition, at the tissue level, the dramatic reduction of nutrients correlated with calorie restriction leads to an increase in autophagy, a very efficient way to eliminate old and noxious molecules. It has been largely reported that the pathways associated with nutrient-sensing signaling, such as IGF (insulin-like growth factor)/insulin and TOR (target of rapamycin), have a key role in this process. Since these strategies have recently been studied also in primates and humans and shown promising results, it has been proposed to search for molecules mimicking the effects of calorie restriction without the side effects that the dramatic reduction of nutrient intake may have on humans (such as depression). To this end, the lack of specific amino acids in the diet and the use of rapamycin are particularly promising interventions to extend health span. Moreover, encouraging studies are currently carried out on the capacity of spermidine to promote autophagy, improve the health of the organism, and, consequently, prolong lifespan.

In the present issue, we have included a number of original articles and updated reviews covering different areas of studies in the field of healthy ageing and longevity. Most of these reports highlight different aspects of the importance of efficient tissue maintenance, especially against oxidative stress (see the papers by M. Ingles et al., S. Heinzel et al., I. Sadowska-Bartosz and G. Bartosz, B. Arosio et al., G. R. Hunter et al., and T. A. Giancaspero et al.). G. Taormina and M. G. Mirisola give an updated review of the effects of calorie restriction on longevity and some possible interventions, taking advantage of the pathways highlighted by studying calorie restriction. The papers by A. Galán-Mercant and A. I. Cuesta-Vargas and S. Garasto et al. show how the close monitoring of health and physical functioning may help in understanding the interventions which may help the quality of human aging. Finally, the paper by P. Garagnani et al. shows the most recent results on the genetic component of longevity and healthy aging suggesting they come from the interactions of three genetic systems.

On the whole, we believe these papers may help the readers have an idea of the different facets of the studies on aging and longevity and of the perspectives they are unveiling.

*Giuseppe Passarino* *Giuseppina Rose* *Dina Bellizzi* *Maria De Luca* *Efstathios S. Gonos*

